# Pre- and Postnatal Exposure to Moderate Levels of Ethanol Can Have Long-Lasting Effects on Hippocampal Glutamate Uptake in Adolescent Offspring

**DOI:** 10.1371/journal.pone.0127845

**Published:** 2015-05-15

**Authors:** Giovana Brolese, Paula Lunardi, Daniela F. de Souza, Fernanda M. Lopes, Marina C. Leite, Carlos-Alberto Gonçalves

**Affiliations:** 1 Department of Neuroscience, Basic Science Health Institute, Federal University of Rio Grande do Sul—UFRGS—Porto Alegre, Rio Grande do Sul, Brazil; 2 Department of Biochemistry—Basic Science Health Institute—Federal University of Rio Grande do Sul—UFRGS—Porto Alegre, Rio Grande do Sul, Brazil; Radboud University, NETHERLANDS

## Abstract

The developing brain is vulnerable to the effects of ethanol. Glutamate is the main mediator of excitatory signals in the brain and is probably involved in most aspects of normal brain function during development. The aim of this study was to investigate vulnerability to and the impact of ethanol toxicity on glutamate uptake signaling in adolescent rats after moderate pre and postnatal ethanol exposure. Pregnant female rats were divided into three groups and treated only with water (control), non-alcoholic beer (vehicle) or 10% (v/v) beer solution (moderate prenatal alcohol exposure—MPAE). Thirty days after birth, adolescent male offspring were submitted to hippocampal acute slice procedure. We assayed glutamate uptake and measured glutathione content and also quantified glial glutamate transporters (EAAT 1 and EAAT 2). The glutamate system vulnerability was tested with different acute ethanol doses in naïve rats and compared with the MPAE group. We also performed a (lipopolysaccharide-challenge (LPS-challenge) with all groups to test the glutamate uptake response after an insult. The MPAE group presented a decrease in glutamate uptake corroborating a decrease in glutathione (GSH) content. The reduction in GSH content suggests oxidative damage after acute ethanol exposure. The glial glutamate transporters were also altered after prenatal ethanol treatment, suggesting a disturbance in glutamate signaling. This study indicates that impairment of glutamate uptake can be dose-dependent and the glutamate system has a higher vulnerability to ethanol toxicity after moderate ethanol exposure *In utero*. The effects of pre- and postnatal ethanol exposure can have long-lasting impacts on the glutamate system in adolescence and potentially into adulthood.

## Introduction

The toxic effects of ethanol are one of the main causes of injury to the developing brain. The negative consequences of prenatal ethanol exposure (PEE) are not limited to high levels of alcohol [1], since moderate PEE can cause brain damage in offspring that is associated with life-long behavioral, social and cognitive disorders [2,3].

Nearly 50% of women report drinking alcohol prior to pregnancy recognition [1,4,5] and 18% continue to drink even after becoming aware of their pregnancy [6,7]. Rates of maternal alcohol consumption are variable among nations; many European countries report very high rates of drinking during pregnancy [7]. Our previous study with an animal model of moderate drinking during pregnancy indicated that prenatal ethanol-exposed offspring exhibit increased risk-taking behaviour and increased N-methyl-D-aspartate receptor—NMDA receptors [8]. These effects appear to be temporally specific and involve stage-specific changes in cell function or gene regulation. Therefore, depending on the time and levels of ethanol exposure, ethanol could interfere with cell proliferation, migration and function, affecting the balance of synaptic plasticity and support from glial functions [9].

The hippocampus is an area of the brain that is highly vulnerable to the effects of alcohol during development [10]. Astrocytes envelop the tripartite synapse and fulfill a range of important and essential functions, including extracellular ion homeostasis, metabolic supply to neurons and the modulation of synaptic transmission and synaptic plasticity [11]. They also support the blood—brain barrier and help maintain glutathione (GSH) levels in the brain [12,13]. Astrocytes are more resistant than other neural cells to oxidative stress and provide a protective role for neurons, mainly due to their higher GSH content. Hence, the cell’s ability to reduce or synthesize GSH is an important key factor that may determine how the cells manage oxidative stress induced by neurotoxicity [14].

While it is well established that dopaminergic, glutamatergic and GABAergic neurotransmission plays an important role in alcohol addiction, the case for chronic and moderate alcohol exposure during pregnancy is still debatable [15]. Glutamate is the main mediator of excitatory signals in the mammalian central nervous system (CNS) and is probably involved in most aspects of normal brain function including cognition, memory and learning [16]. It also plays major roles in the development of the CNS, including synapse induction and elimination, and cell migration and differentiation. Both neurons and astrocytes have glutamate receptors in their plasma membranes [17,18] and one of the main functions of astrocytes is glutamate uptake from the tripartite synapse. Glutamate uptake is the mechanism responsible for the long-term maintenance of low extracellular concentrations of glutamate and it is accomplished by means of glutamate transporter proteins [19,20].

Astrocytes express both excitatory amino acid transporters, EAAT 1 and EAAT 2, to remove glutamate from the extracellular fluid. Thus, they can modulate neurotransmission by maintaining low concentrations of extracellular glutamate and preventing accumulation of toxic concentrations. Acute and high doses of ethanol increases glutamate uptake activity [21] and repeated bouts of moderate ethanol consumption alter basal glutamate dynamics in hippocampus. Depending on the dose administered, acute ethanol exposure has been reported to either increase, decrease or have no effect on glutamate levels in the hippocampus [22].

While the ability of ethanol to interact with glutamatergic pathways and NMDA receptors is well established, relatively little is known about its ability to modulate endogenous uptake of glutamate following pre- and postnatal exposure to a moderate dose of ethanol. Furthermore, astrocytes are well known to play a pivotal role in CNS immunity [23,24] and some studies have demonstrated that chronic ethanol consumption increases cytokines and inflammatory mediators in the rat brain, activating signaling pathways associated with neuroinflammation and triggering cell damage [25]. In addition, ethanol immunomodulatory effects can result in suppression of the general immune system as well as defense against microbial and viral infections [26].

The aim of this study was to investigate vulnerability to and impact of ethanol toxicity on glutamate uptake signaling in adolescent offspring after moderate pre- and postnatal ethanol exposure. The study used an alcoholic beer solution instead of ethanol in water to increase palatability and ethanol intake by rats [27–29]. The ‘beer model’ allows self-administration of ethanol without stressing the animal with injection, intubation or a forced diet [30]. This model was employed as an experimental protocol for standard pregnant female laboratory rats that consumed moderate amounts of alcohol during pregnancy and lactation. The offspring’s biochemical analyses allowed us to check the vulnerability of pre- and postnatal ethanol exposure especially on glutamate uptake system.

## Materials and Methods

### Animals

Female Wistar rats (n = 24), initially weighing 190–240g were maintained on a 12:12-hour light/dark cycle (7:00 a.m. 7:00 p.m. light phase). The room temperature was held constant (21–22°C). All animals were given *ad libitum* access to water and standard rat chow (Nuvital Nutrientes S/A, Colombo, Brazil) throughout the study.

Females were mated during the dark phase of their oestrous cycle. In the following morning, on the light cycle, we checked if spermatozoa were present in the vaginal smears using 0.9% sodium chloride (NaCl). The gestational day one (GD 1) was defined when sperm were detected. If pregnancy was confirmed the dam was immediately housed individually and assigned to one of three groups: only water (n = 8, control group), non-alcoholic beer (n = 8, vehicle group) or 10% beer solution (n = 8, moderate prenatal alcohol exposure [MPAE] group: non-alcoholic beer + 10% v/v ethanol). Rat chow and water were available *ad libitum* until the end of the study in all groups. Beer solution was available throughout gestation and until weaning, on postnatal day (PND) 22. During postnatal period, the offspring were exposed to ethanol solution only by suckling their dams. Until PND 22 they were not tall enough to reach the beer bottles. Usually, during lactation period, the dams increase the fluids intake, which means a higher amount of alcohol consumption (see Table 1). Only adolescent male offspring (PND 30) were used for the subsequent analysis. To minimize the influence of the litter effect, we collected only two male offspring from each litter, and multiple litters were used for each analysis. All animal use and care procedures were in accordance with the National Institutes of Health guidelines and were approved by the Federal University of Rio Grande do Sul Animal Care Committee.

**Table 1:** Fluid/food consumption of the dams and developmental data from offspring.

Groups	Control		Vehicle		MPAE	
	Pregnancy	Lactation	Pregnancy	Lactation	Pregnancy	Lactation
**Parameter**	0.00	0.00	32.3 ± 1.02	27.3 ± 2.8	27.5 ± 1.8	42.7 ± 3.9**
Beer solution intake (mL)						
Water intake (mL)	53.9 ± 4.1	140 ± 8.7	35.9 ± 8.1	118.9 ± 11.1	39.1 ± 2.4*	83.2 ± 20.9*
Food intake (g)	25.3 ± 1.4	26.4 ± 1.5	29.3 ± 0.8	28.7 ± 5.1	30.7 ± 1.4	24.05 ± 1.4
BAC (mg/dL)					139.4 ± 30.04	
Litter (n)	n = 10.4 ± 0.3		n = 11.02 ± 0.5		n = 9.08 ± 0.7	
Offspring Weight (g)						
PND 2	7.68 ± 0.1		7.76 ± 0.2		7.78 ± 0.1	
PND 7	14.3 ± 0.3		13.85 ± 0.2		14.06 ± 0.3	
PND 12	21.3 ± 0.6		21.64 ± 0.4		20.9 ± 0.4	
PND 17	30.5 ± 0.7		31,71 ± 0.6		32.62 ± 0.7	
PND 22	39.4 ±1.2		41.15 ± 1.5		42.35 ± 1.1	

*The MPAE group is different from the control group, p < 0.05 and **the MPAE group is different from the vehicle group, p < 0.05. Data are mean ± SEM.

### Treatment

Treatments were as described by [8]. Briefly, non-alcoholic beer containing water, malt, carbohydrates, an antioxidant (INS316), a stabilizer (INS305) and hops (manufacturer’s information) was thoroughly decarbonated by pouring into a 2 L beaker, and was kept under continuous agitation for 50 min before use. Fresh beer solutions made with ethanol purchased from Merck (Darmstadt, Germany) were delivered daily at room temperature from GD 1 throughout gestation until weaning (PND 22). Note that dams from the MPAE and vehicle groups had limited access to beer solution (50 mL per day) in order to control the caloric intake. The ethanol dose of 10% v/v ethanol was chosen based on the fact that studies using low to moderate doses reach BACs of 80–150 mg/dl and animal models of binge drinking and/or fetal alcohol syndrome (FAS) usually reach BACs >200 mg/dl, although there is no agreed guidelines in the literature defining the exactly amounts of low, moderate nor high ethanol exposure, we carefully considered a dose containing a lower amount of alcohol than FAS [31]. Every day at 4:00 p.m., the bottles of beer solution were exchanged for fresh ones. Two bottles were provided, one with beer and the other with water, and the bottles’ positions were alternated. The control group received water *ad libitum* as the only source of drink.

### Hippocampal Acute Slices Preparation

Hippocampal slices were prepared as previously described by Nardin [32]. Briefly, on PND 30, male offspring Wistar rats from control, vehicle and MPAE groups were killed by decapitation. The brains were removed and placed in cold saline medium with the following composition (in mM): 120 NaCl, 2 KCl, 1 CaCl_2_, 1MgSO_4_, 25 HEPES, 1 KH_2_PO_4_, 10 glucose adjusted to pH 7.4 and previously aerated with O_2_. The hippocampi were dissected and transverse slices of 0.3 mm were obtained using a McIlwain Tissue Chopper. Slices were immediately transferred to 24-well plates, each well containing only one slice and 0.3 mL of HBSS medium, containing (in mM): 137 NaCl, 5.36 KCl, 1.26 CaCl_2_, 0.41 MgSO_4_, 0.49 MgCl_2_, 0.63 Na_2_HPO_4_.7H2O, 0.44 KH_2_PO_4_, 4.17 NaHCO_3_, and 5.6 glucose, adjusted to pH 7.4. The medium was changed every 15 min for 2 h at room temperature. After this stabilization period, medium was collected for S100B secretion analyses and slices were used for the following four techniques described.

### Analyses *in vitro*

Hippocampal slices were prepared for a dose-curve with different concentrations of ethanol (5 mM, 50 mM and 500 mM), according to previous studies [33,34]. Thirty-day-old naïve male Wistar rats and male offspring from the MPAE group were killed by decapitation. After the stabilization period of hippocampal acute slices preparation we did 1h-treatment with different ethanol concentrations followed by glutamate uptake analysis.

### Glutamate Uptake Assay

After the stabilization period, the medium was replaced by Hank’s balanced salt solution (HBSS), containing (in mM): 137 NaCl, 5.36 KCl, 1.26 CaCl_2_, 0.41 MgSO_4_, 0.49 MgCl_2_, 0.63 Na_2_HPO_4_.7H2O, 0.44 KH_2_PO_4_, 4.17 NaHCO_3_, and 5.6 glucose, adjusted to pH 7.4. The slices were maintained at 35°C, and the assay was started by adding 0.1mM L-glutamate and 0.66 μCi/mL L-[2,3-3H]-glutamate, both obtained from Amersham (United Kingdom); this high unlabeled glutamate concentration renders the assay insensitive to changes in glutamate release. Incubation was stopped after 5 min by removing the medium and rinsing the slices three times with ice-cold HBSS. Slices were then lysed in a solution of 0.5mM MNaOH. Sodium-independent (nonspecific) uptake was determined using a solution with N-methyl-D-glutamine instead of NaCl. Sodium-dependent glutamate uptake was obtained by subtracting nonspecific uptake. Radioactivity was measured with a scintillation counter (2800TR TriCarb Liquid Scintillation Analyzer, Perkin-Elmer, Waltham, MA, USA). Final glutamate uptake was expressed as nmol/mg protein/min.

### Glutathione (GSH) Content Assay

Glutathione levels (nmol/mg protein) were measured as previously described by [35]. Cell homogenates were diluted in ten volumes of 100mM sodium phosphate buffer, pH 8.0, containing 5 mM EDTA, and protein was precipitated with 1.7% meta-phosphoric acid. The supernatant was assayed with o-phthaldialdehyde (1 mg/mL methanol) at room temperature for 15 min. Fluorescence was measured by using excitation and emission wavelengths of 350nm and 420 nm, respectively. A calibration curve was constructed with standard GSH solutions (0–500μM).

### Electrophoresis and Western Blot Analysis

Hippocampal slices for electrophoresis/Western blot analysis were directly homogenized in electrophoresis sample buffer at pH 6.8 (containing 62.5 mM Tris-HCl, 2% (w/v) SDS, 5% (w/v) β-mercaptoethanol, 10% (v/v) glycerol, 0.002% (w/v) bromophenol blue) and boiled for 2 min. Protein samples (20μg per lane) were analyzed by 10% sodium dodecyl sulfate polyacrylamide gel electrophoresis (SDS-PAGE) and transferred to nitrocellulose membranes using a semidry blotting apparatus (1.2mA/cm^2^, 1 h; [36]. The membranes were blocked overnight with 5% bovine serum albumin (BSA) in Tris-buffered saline (TBS; 10mM Tris, 150mM NaCl, pH 7.5) and then incubated for 3 h with an anti-EAAT 1 and anti-EAAT 2 (antibody diluted 1:1000 and 1:200, respectively, in TBS containing Tween-20 and 2% BSA). Next, membranes were incubated for 2h at room temperature with horseradish peroxidase (HRP)-conjugated anti-mouse secondary antibody (GE Healthcare, Sao Paulo—Brazil). The chemiluminescent reactions were developed using luminol as the substrate (ECL Western Blotting System, GE Healthcare, Sao Paulo—Brazil) and registered on radiographic film. The immune content of EAAT 1 and EAAT 2 was determined as a ratio of the optical density (OD) of the β-actin protein band [(EAAT1/2)/OD of the β-actin band]. The bands were quantified using Scion Image software.

### Protein Content

The total protein content was determined by the modified method of Lowry [37], using BSA as the standard.

### Statistical Analysis

Parametric data are reported as mean ± standard error and were analyzed by one-way ANOVA or two-way-ANOVA followed by Tukey’s or Newman-Keuls post-hoc, respectively. Significance level was set at p < 0.05. Statistical analyses were performed with GraphPad Prism 6 for OS-X (GraphPad Inc, La Jolla, CA, USA).

## Results

Data on alcohol consumption, offspring weight gain and blood ethanol levels (BEL) are summarized in Table 1. BEL was determined only from dams in the MPAE group by collecting blood following decapitation. BEL was quantified using the Sigma diagnostic kit (332UV) according to the manufacturer’s instructions.

### Glutamate Uptake

**Fig 1:**
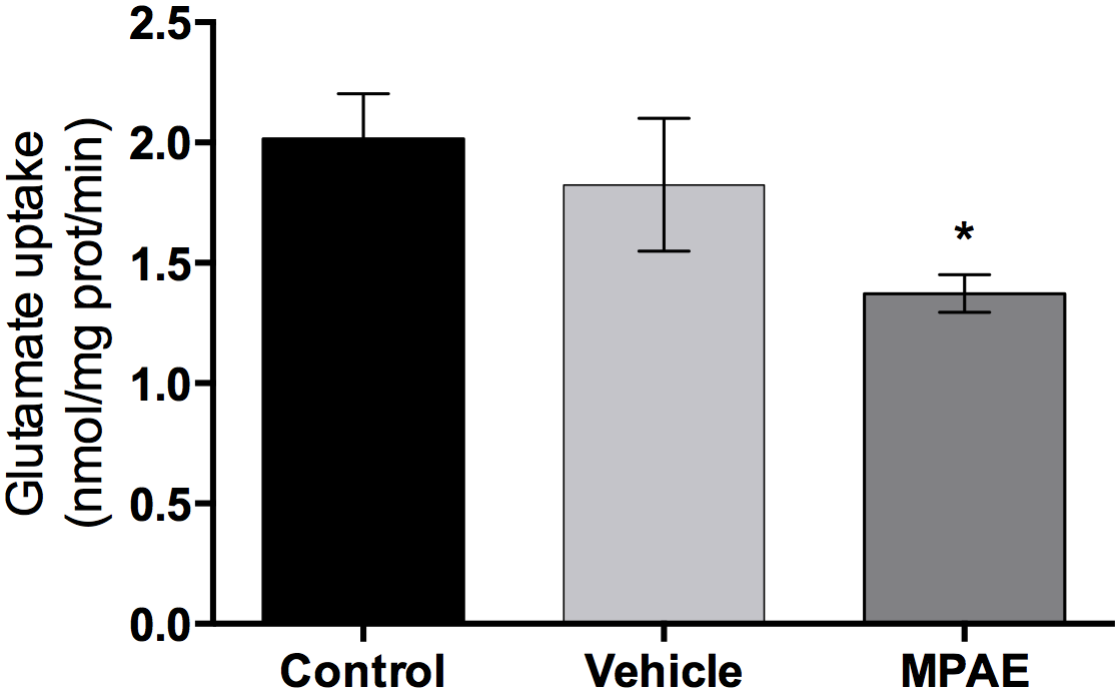
Glutamate uptake. Hippocampal slices of offspring on PND 30 after beer solution treatment. Each value represents mean ± SEM of ten independent experiments performed in triplicate. One-way ANOVA followed by Tukey’s post-hoc test showed that the MPAE group was significantly different from the control group, * p = 0.022, (SE = 0.212), but not different from vehicle group (p = 0.180; SE = 0.240). Also, control group was not different from vehicle (p = 0.737; SE = 0.253).

The results of the glutamate uptake assay are shown in Fig 1. One-way ANOVA revealed a decrease in glutamate uptake for MPAE group [F (2,15) = 4.881, p = 0.023] and Tukey post-hoc showed that MPAE group was statistically different only from control group (p = 0.022). We found a reduction in glutamate uptake only for MPAE group, and we tested whether different ethanol doses altered the vulnerability of the glutamate system to ethanol toxicity.

**Fig 2:**
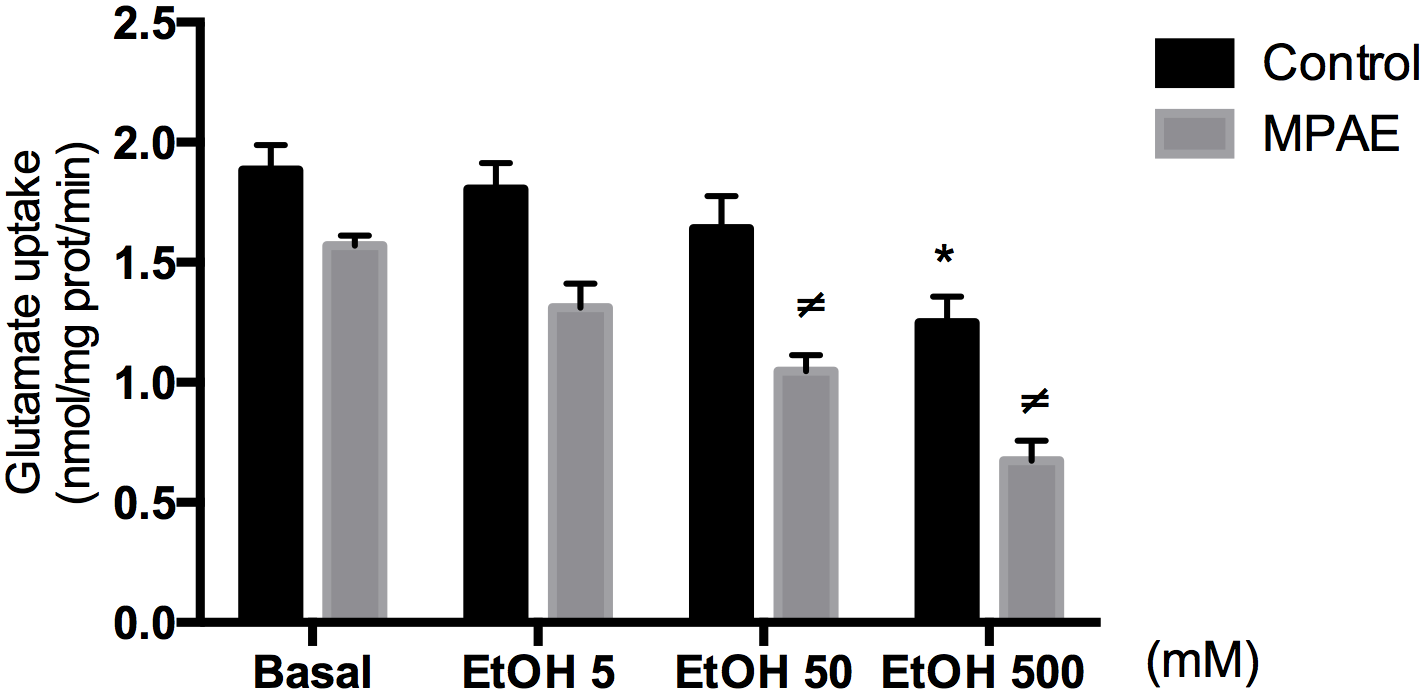
Glutamate uptake after ethanol treatment. Hippocampal slices of Control (naïve) and MPAE rats were treated with different ethanol concentrations. Each value represents mean ± SEM of three experiments performed in triplicate. Two-way-ANOVA followed by Newman-Keuls post-hoc revealed that there are significant differences between and within groups after treatment with different concentrations of ethanol. In Control group only basal was different from EtOH 500 (*p = 0.001). In MPAE group the basal was different from EtOH 50 (^≠^p = 0.006) and EtOH 500 (^≠^p = 0.0001). Still in MPAE group, EtOH 5 was different from EtOH 500 (p = 0.001). Analysis between groups revealed that in both EtOH 50 and EtOH 500 concentrations, Control and MPAE groups were statistically different (p = 0.002; p = 0.001, respectively).

The glutamate uptake for *in vitro* test used hippocampal acute slices from a group of thirty-day-old naïve male rats (Control group) and from MPAE group. A two-way-ANOVA was performed with treatment (5 mM, 50 mM or 500mM of ethanol-95%) as the between-subjects factor and groups (Control and MPAE) as the within subject factor. There was a significant treatment [F (3,25) = 22.71, p < 0.0001] and group effects [F (1,25) = 48.13, p < 0.0001], but no interaction (Fig 2). A Newman-Keuls post-hoc test revealed that in Control group only the EtOH 500 mM treatment differed from the baseline (no ethanol) (p = 0.001). However, in MPAE group, a significant reduction in glutamate uptake occurred not only in the highest concentration of ethanol (500 mM; p = 0.0001), but also in the 50 mM EtOH concentration (p < 0.006), when compared to baseline. Still in MPAE group, EtOH 5 was different from EtOH 500 (p = 0.001). Analysis between groups revealed that in both EtOH 50 and EtOH 500 concentrations, Control and MPAE groups were statistically different (p = 0.002; p = 0.001, respectively; Fig 2).

**Fig 3:**
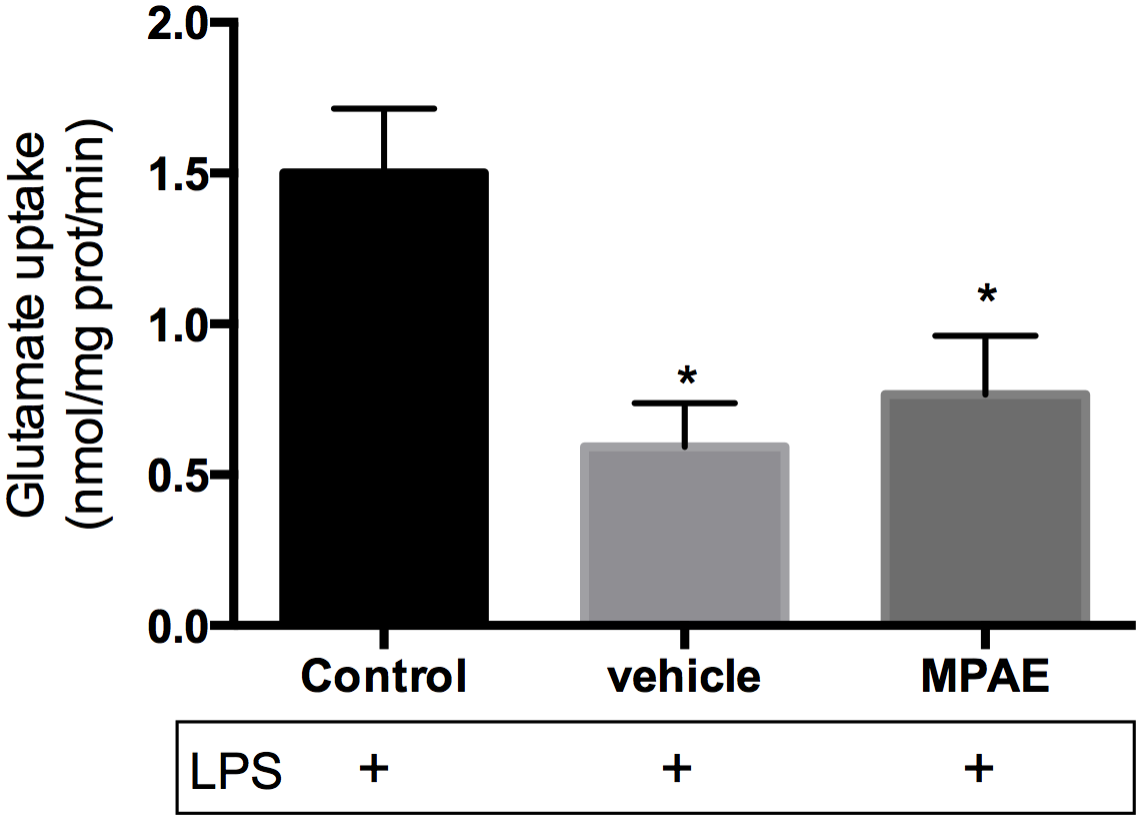
Glutamate uptake after LPS-Challenging. Glutamate uptake in hippocampal slices of offspring on PND 30 was measured after LPS insult. Each value represents mean ± SEM of four experiments performed in triplicate. One-way ANOVA followed by Tukey’s post-hoc test showed that both the vehicle (p = 0.016 and SE = 0.218) and MPAE groups (p = 0.003 and SE = 0.166) were different from the control group. + means the presence of LPS.

To further investigate the influence of alcohol treatment in the MPAE group on glutamate uptake activity we challenged the hippocampal slices with an acute administration of LPS at 0.1 μg/mL for 1 hour (Lipopolysaccharides from *Escherichia coli* (LPS) 055:B5 were purchased from Sigma (St. Louis, USA). Soon after, we measured the glutamate uptake response (Fig 3). One-way-ANOVA showed a significant difference among groups [F (2,14) = 12.37, p = 0.0008] and Tukey’s post-hoc showed that both vehicle (p = 0.016) and MPAE (p = 0.003) groups were different from the control group, suggesting that groups that received beer solution (with or without ethanol) were more vulnerable to glutamate uptake reduction.

### Glutathione (GSH) Content

**Fig 4:**
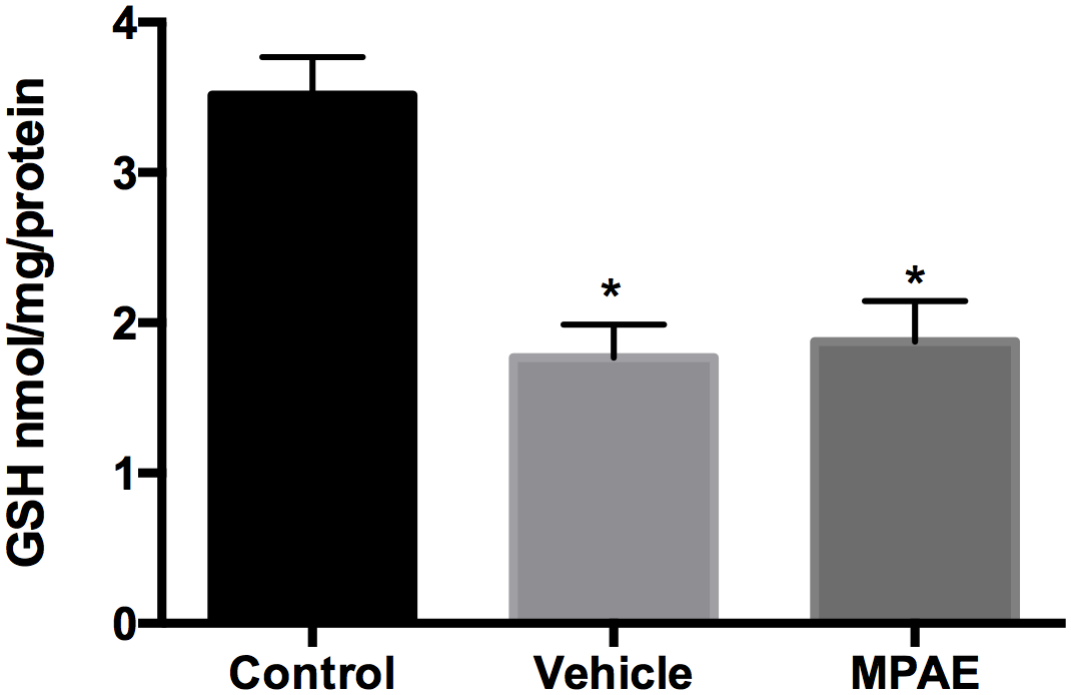
Glutathione content. Hippocampal slices of offspring on PND 30 after beer solution treatment. Each value represents mean ± SEM of five experiments performed in triplicate. One-way ANOVA followed by Tukey’s post-hoc test showed that both vehicle (p = 0.009 and SE = 0.501) and MPAE groups (p = 0.007 and SE = 0.457) are different from the control group.

To investigate astroglial response we measured the intracellular content of glutathione in hippocampal slices after prenatal treatment with beer solution. One-way-ANOVA showed a significant decrease in GSH content in both MPAE and vehicle groups [F (2,14) = 7.43, p = 0.006), and Tukey’s post-hoc test revealed that both groups were different from the control group (vehicle p = 0.009; MPAE p = 0.007), suggesting that groups that consumed non-alcoholic or alcoholic beer showed differences related to a stress response (Fig 4).

### Western Blot Analysis

**Fig 5:**
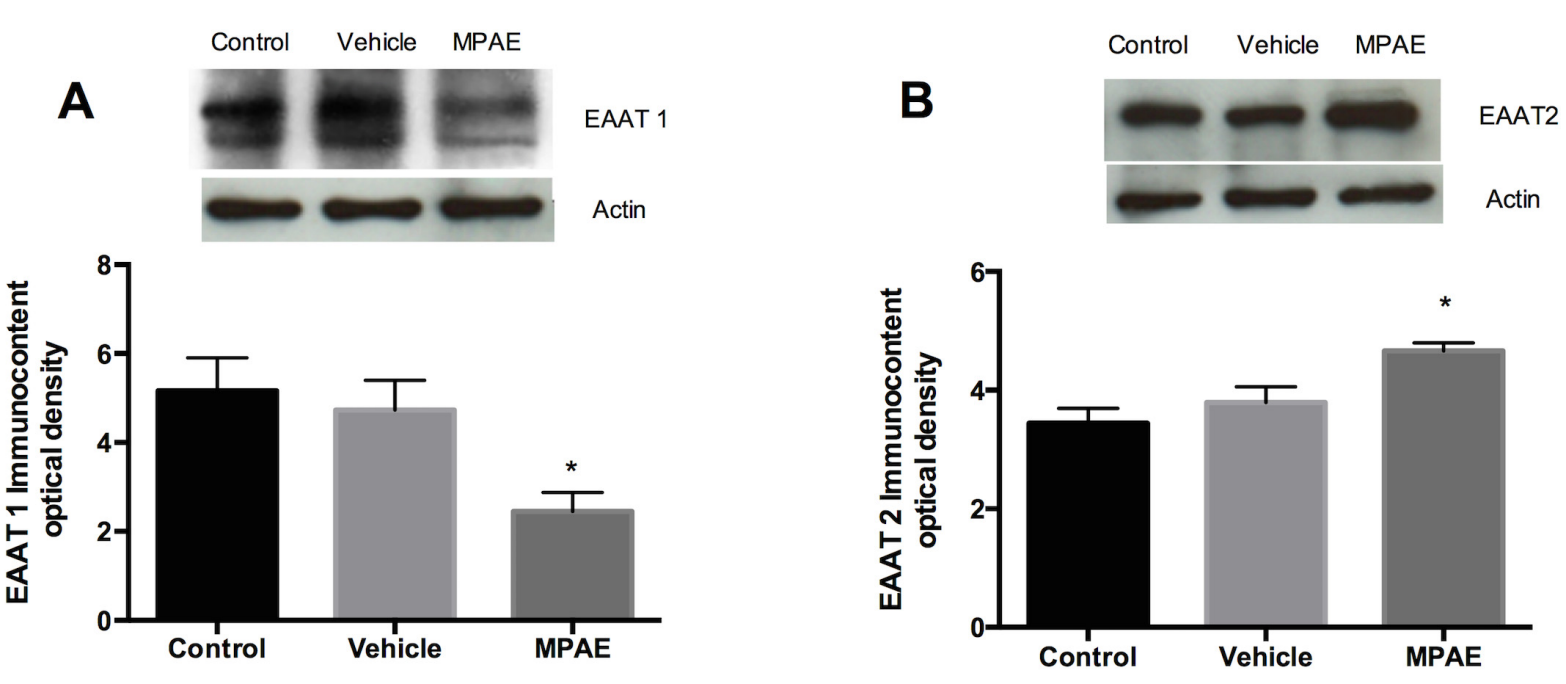
EAAT 1 and EAAT 2 immunoquantification. The hippocampus was dissected and homogenized for the measurement of EAAT 1 and EAAT2 transporters by Western blot analysis. Values are mean ± SEM for three rats per group. **A**. One-way ANOVA revealed that EAAT 1 was decreased and Tukey’s post-hoc test showed that the MPAE group was different from control (p = 0.010 and SE = 0.895) and vehicle groups (p = 0.037 and SE = 0.988). **B**. One-way ANOVA revealed that EAAT 2 was increased and Tukey’s post-hoc test showed that the MPAE was different from the control group, * p = 0.016 and SE = 0.317

The immunocontent of EAAT 1 and EAAT 2 glutamate transporters was altered after ethanol treatment (Fig 5). After analysis by one-way-ANOVA, the MPAE group showed a decrease in EAAT 1 expression [F (2,25) = 3.779, p = 0.036]. Tukey post-hoc showed that MPAE differed significantly from the control (p = 0.010) and vehicle (p = 0.037) groups. However, EAAT 2 expression was increased [F (2,60) = 7.790, p = 0.021] only in the MPAE group compared to the control group (p = 0.016). These results suggest that there are differences between EAAT1 and EAAT2 quantification in the same group (MPAE); and further studies will be needed to evaluate localization and functionality of the transporters.

## Discussion

Our study revealed the effects of moderate PAE on glial parameters, especially on the glutamate system. Our data showed a decrease in glutamate uptake, GSH content and a significant change in level of expression of glutamate transporters after pre- and postnatal ethanol exposure in hippocampal slices of adolescent male rats. These changes in glial parameters suggest astroglial vulnerability to ethanol during brain development, even at a moderate dose.

Prenatal ethanol exposure has been shown to increase oxidative stress in the developing brain [38,39]. Glutamate is also one of the three amino acids constituting the glutathione (GSH) structure, which is the main non-enzymatic endogenous antioxidant of glial cells, and has a pivotal role in cellular homeostasis [40]. The cellular ability to synthesize glutathione is an important factor in the management of oxidative stress-induced neurotoxicity. Our results showed a significant decrease in GSH content after beer treatment, not only in the MPAE group but also in the vehicle group, suggesting that maybe non-alcoholic beer also contains components that could interfere in oxidative stress. A compromised GSH system in the brain has been connected with increased oxidative stress occurring during brain injury [35]. Ethanol can lead to an increase in the production of reactive oxygen species (ROS) and/or a decrease in the levels of antioxidant defenses causing a redox imbalance and resulting in the oxidative damage of lipids, proteins, and DNA [41]. GSH has also important extracellular functions in brain. In this respect astrocytes appear to play a key role in GSH metabolism, since astrocytes are the main source of GSH in SNC, the exporting of this antioxidant is essential for providing precursors to neurons [42]. Exposure of rodents to ethanol during pregnancy and in the first days of postnatal life (which are equivalent to the third gestational trimester in humans) has also been shown to alter the GSH content while increasing the levels of lipid peroxides and protein carbonyls in several brain regions [43,44]. Therefore, oxidative stress can contribute, at least in part, to the neurodegeneration observed in fetal alcohol spectrum disorders.

There is evidence that glutamate plays an important role in the neuroadaptations associated with prolonged ethanol exposure [15]. The glutamate system is involved in many aspects of brain function, including development [45]. Our data showed a decrease in glutamate uptake only in animals treated with ethanol (MPAE group), suggesting an alteration in glutamate signaling. Astrocytes have an important functional role in the CNS, since they are responsible for most of the glutamate uptake in the synaptic cleft [16]. Extracellular glutamate levels are maintained through a balance between release and uptake and glutamatergic disturbance can be due to different factors, including failure of glutamate transporters. Some studies have shown that chronic ethanol treatment leads to lower rates of glutamate uptake contributing to an increase in basal extracellular glutamate levels and neurotoxicity [46–48]. Thus, repeated ethanol exposure during brain development may contribute to the hippocampal impairment, even under moderate ethanol doses [34].

Glutamate is released into the synapse and rapidly removed by a family of EAATs localized in neurons and glial cells. We quantified the total tissue content of two glutamate glial transporters (EAAT 1 and EAAT 2), which provide the majority of functional glutamate transport in the CNS [49]. We found a reduction in EAAT 1 transporter expression, suggesting that the glutamate uptake decrease could be, in part, associated with this downregulation. However, EAAT 2 transporter expression was upregulated in the MPAE group, a response generally seen after acute ethanol exposure [50], when an increase in extracellular glutamate also occurs [47,48]. A study by Melendez et al. (2005) found a significant decrease in glutamate uptake in accumbens slices 24 h after 7 days of repeated moderate ethanol exposure. The ethanol-induced deficit in glutamate uptake was not associated with a decrease in total tissue levels of glial glutamate transporters, since glutamate uptake normalized 14 days after discontinuing ethanol exposure [34]. However, the electrophoresis technique is not able to detect the localization and functionality of these transporters, which means that EAAT 2 overexpression may not be specifically related to the cell membrane. Because the transporters are a membrane-bound pump that regulates extracellular glutamate levels, studies using gene expression are required to investigate further the viability and functionality or post-translational modifications of these membrane-transporters.

To test whether the deficit in glutamate uptake/recycling mechanisms could be does-dependent we treated hippocampal acute slices from naïve or MPAE rats with ethanol in different concentrations and measured glutamate uptake activity. Interestingly, we noticed that naïve hippocampal slices showed a decrease in glutamate uptake only at the highest concentration of ethanol (500 mM), but the MPAE group revealed a decrease at both 50mM and 500mM ethanol concentrations. It is interesting to note that the basal glutamate uptake levels on slices from MPAE animals (Fig 3) are similar to the one found on slices from naïve animals treated with 500 mM EtOH (Fig 2). These findings suggest that reduction of glutamate uptake activity can be dose-dependent, mainly when tested acutely. Thus, we observed hippocampal vulnerability to ethanol toxicity effects during fetal development, indicating that these effects could last as long as adolescence.

It is already known that chronic ethanol exposure can induce neuroinflammation and brain damage, mostly through the reaction of astroglial cells [51]. In order to test the vulnerability of glutamate uptake to toxic effects of a moderate ethanol dose, we challenged hippocampal slices with acute administration of LPS followed by a glutamate uptake assay. Our results showed a higher vulnerability in MPAE and vehicle groups compared to the control group, suggesting that LPS insult was enough to impair glutamate signaling in pretreated animals but not in the control group.

Considering the results, we hypothesized that the vehicle group would follow the results of the control group, once there was no alcohol in both solutions offered to these groups. According to the manufacturer’s information, the Liber beer used in this study contains water, malt, carbohydrates, an antioxidant (INS316), a stabilizer (INS305) and hops; and they assumed that this is a 0.0% alcohol beer, different from another brands that state on the label that is non-alcoholic beer, however contain small amounts of alcohol (0.05%). Curiously, the vehicle group also differed significantly from the control group for glutamate uptake after LPS insult and for GSH content, suggesting not only an ethanol effect but also a non-alcoholic beer effect *per se*. We could speculate that the non-alcoholic Liber beer formula contains some element(s) or ingredient(s) that increases the vulnerability to oxidative stress (GSH) and inflammation (LPS). Further analytical tests analyzing the compounds of non-alcoholic beer are needed to clarify this issue. Very few studies have used the beer model during the prenatal and postnatal period, suggesting that administration of non-alcoholic beer *in utero* may result in unexpected effects [52,53]. Moreover, some (non-ethanol) components of non-alcoholic beer could be responsible for the effects found. Further studies are needed to determine the effects of the components, since the beer used in these experiments was not tested for congeners. Evidence from other studies indicates that beer contains many compounds such as lead, nitrosamines, phytoestrogens and small amounts of alcohol which could interfere with results [53].

Our data suggests that alterations in the glutamatergic system appear to contribute to fetal alcohol vulnerability during pregnancy, and novel glutamatergic agents may play a key role in the treatment of ethanol abuse and dependence. However, findings may vary due to differences in animal models and ethanol doses, as well as the brain region studied. The threshold dose of ethanol capable of producing glutamate system impairment is likely to vary, but even moderate doses during gestation can be harmful to the fetal brain. The decrease in GSH and glutamate uptake show a great vulnerability to ethanol-induced effects associated with the oxidative stress response and impairment in glial glutamate transporters during development. Moreover, we showed that the effects of prenatal ethanol exposure can be observed in the glutamate system in adolescence and may last up to adulthood.
